# Assessing national cervical cancer screening guidelines: Results from an HIV testing clinic also screening for cervical cancer and HPV in Soweto, South Africa

**DOI:** 10.1371/journal.pone.0255124

**Published:** 2021-07-30

**Authors:** Kathryn L. Hopkins, Maya Jaffer, Khuthadzo E. Hlongwane, Kennedy Otwombe, Janan Dietrich, Mireille Cheyip, Jacobus Olivier, Tanya Doherty, Glenda E. Gray

**Affiliations:** 1 Perinatal HIV Research Unit, Faculty of Health Sciences, University of the Witwatersrand, Chris Hani Baragwanath Academic Hospital, Johannesburg, South Africa; 2 School of Public Health, Faculty of Health Sciences, University of the Witwatersrand, Johannesburg, South Africa; 3 Health Systems Research Unit, South African Medical Research Council, Cape Town, South Africa; 4 Centers for Disease Control and Prevention, Pretoria, South Africa; 5 Office of the President, South African Medical Research Council, Cape Town, South Africa; Fred Hutchinson Cancer Research Center, UNITED STATES

## Abstract

**Objective:**

A screening centre in Soweto, South Africa (SA), investigated high-risk human papillomavirus (HR-HPV), HIV, cervical cancer risk amongst women.

**Methods:**

This cross-sectional study (June 2018-March 2019) describes screening results (Roche Linear Array HPV test and Pap smear liquid based cytology) and history of screening (known HIV status, antiretroviral therapy [ART] use, previous Pap smears). Data were stratified by age group (18–29, 30+ years), HIV status, Pap smear results and tested for statistical significance.

**Results:**

Of 280 women, 20.4% were HIV-positive, 18.2% had abnormal Pap smears, 41.8% had HR-HPV. Of older women, 48.2% (n = 78/162) had never had a Pap smear. Of younger women, 89.0% (n = 105/118) never had a Pap smear, but had significantly more low-grade squamous intraepithelial lesions (LSIL) and other HR-HPV infection than older women (12.7%[n = 15/118] vs 4.9%[n = 8/162], p = 0.0193; and 49.2%[n = 58/118] vs 29.0%[n = 47/162], p = 0.0006; respectively). HIV-positive women had more abnormal cytology results and infection with other HR-HPV types or co-infection with other HR-HPV type(s)/HPV-16 compared to HIV-negative women (35.1%[n = 20/57] vs 13.9%[n = 31/223], p = 0.0002; 56.1%[n = 32/57] vs 32.7%[n = 73/223], p = 0.001; and 12.3%[n = 7/57] vs 4.9%[n = 11/223], p = 0.044; respectively). Of 57 HIV-positive women, 45.6% (n = 26) already knew their HIV status; of which 69.2% were on ART and 34.6% never had a Pap smear.

**Conclusion:**

South African women have high rates of HIV, Pap smear abnormalities and HR-HPV, with low cervical cancer screening coverage. SA cervical cancer screening policy excludes (undiagnosed) HIV-positive and HIV-negative women <30 years, both populations found to have high prevalence of HR-HPV. HPV-based primary screening from 25 years could improve outcomes.

## Introduction

In South Africa, cervical cancer is the most common cancer in women aged between 15–44 years [1], as well as the number one cause of cancer deaths among women of all ages [2]. High-risk human papillomavirus (HR-HPV) types 16 and 18 cause approximately 70% of the world’s cases of cervical cancer [1], and they are by far the most common types causing cancer in sub-Saharan Africa. Additionally, other carcinogenic HR-HPV types—such as HPV-35—may be more common in this region than in other parts of the world [3–5].

South Africa is the global epicentre of the HIV/AIDS epidemic, with an estimated 7.9 million people living with HIV (PLHIV) as of 2017 [6–9]. PLHIV are known to have a higher prevalence of HPV infection than their HIV-negative counterparts [4]. This is due to shared risk factors, including sexual behaviour patterns, as well as underlying changes in immune responses during HIV infection [3]. As such, studies have reported cervical cancer as a main disease affecting HIV-positive women residing within low and middle-income countries (LMICs) [3, 4].

Effective public health strategies addressing the burden of HR-HPV infection and cervical cancer in South Africa are vital. One such strategy in the primary prevention of cervical cancer is HPV school-based vaccination amongst girls aged 9–12 years (a bivalent vaccine [targeting HPV 16/18] with a 2-dose schedule [six months apart]) [10, 11]. Since its implementation in 2014, early indications are that vaccine coverage and uptake are high (86.6% vaccinated with the first dose) [10]; however, the uptake between the first and second doses declines by about 25% [10]. Literature suggests parental vaccine hesitancy for the HPV vaccine might be higher than for other childhood vaccines [12, 13]. Long-term sustained effort should be considered to facilitate future successful implementation of HPV vaccination [10, 11]. Beyond vaccination efforts, current cervical cancer screening guidelines issued by the South African National Department of Health (SANDoH) recommend the provision of screening via Pap smear at intervals to the general population of low-risk women who are asymptomatic and HIV-negative. This includes three screening tests within a lifetime performed every 10 years, starting at 30 years of age until 50 years, as the onset of cervical cancer before 30 years is rare [11]. If an abnormality is detected, repeat screenings, including any required treatment, are recommended at three-yearly intervals until a screen result is negative [14].

The SANDoH guidelines recommend screening tests are conducted at a minimum of every three years from the time of diagnosis for HIV-positive women [11]. Typically, these screenings are cytology-based (i.e.; Pap smears). However, in 2017, the national policy allowed for a scaled-up approach to HPV-DNA testing based on the availability of resources at a provincial level [11]. HPV-based primary screening, as advocated for by the South African HPV Advisory Board, is supported by current literature and global practice [15]. HPV testing has a greater sensitivity in detecting precancerous lesions as compared to cytology, and allows for longer intervals between screening tests in the instance of negative screening results [14].

A systematic review has highlighted the lack of evidence on how best to implement systemic cervical cancer prevention in LMICs with high burdens of HIV, including opportunities for and potential benefits of “one-stop shops” for both HIV and cervical cancer services [16]. However, the South African National HIV testing services (HTS) Guidelines [17] do not include screening for either HPV or cervical cancer in the eventuality of a diagnosis of HIV-infection.

An HTS Centre in Soweto, South Africa, expanded its standard of care HTS services for all walk-in adults to also include HR-HPV genotyping and cervical cancer screening via Pap smear liquid based cytology for all female clients. This study investigates the prevalence of HIV, abnormal cytology results and HR-HPV infection; as well as previous uptake of cervical cancer screening among women attending the integrated testing services centre. Screening for HPV and cervical cancer, regardless of HIV status, allowed us to examine the burden of disease by age group and HIV-infection.

## Materials and methods

### Study design, setting and sampling

This was a cross-sectional study describing the health screening results of walk-in adult women attending an integrated health screening clinic at the Perinatal HIV Research Unit (PHRU), a leading research centre situated at the Chris Hani Baragwanath Academic (Bara) Hospital in Soweto, South Africa. The study sample comprised all eligible walk-in female clients, who consented for both the health screening programme and for their data to be captured and used in research between 18 June 2018 and 28 March 2019. If consent was not given, the client was still able to undergo health screening without data capture.

### Inclusion and exclusion criteria

Eligible participants had to be at least 18 years old; fluent in either English, IsiZulu, and/or Sesotho; and provide either written or verbal informed consent (with an impartial witness) for health screening procedures. A client was excluded from the programme and redirected to the Bara casualty ward if she presented to the clinic and there was immediate cause for concern regarding her health.

### Ethical considerations

The health programme was approved by the University of Witwatersrand, Human Research Ethics Committee prior to any data collection. This programme was reviewed and approved by the Associate Director for Science, Center for Global Health, Centers for Disease Control and Prevention (CDC) and was determined to be research, but investigators did not interact with human subjects or have access to identifiable data or specimens for research purposes.

### Data collection and management

Client demographics were either self-collected by literate clients in the reception area or collected with help of the clinic staff for clients unable to read or write. Clinical data, including previous cervical cancer screening uptake and whether HIV-positive clients were newly diagnosed or already knew their status, were collected by the study counsellors and nurse. All data were collected through questionnaires and screening results and recorded directly onto paper forms comprising the PHRU HTS client file. The data were then captured in the study’s electronic database, REDCap. Source documents included the HTS client file and pathology reports, which were printed and filed within the HTS client file. Results from HPV genotyping via Roche Linear Array HPV test and Pap smear liquid based cytology (both analyses conducted using a single swab specimen) were emailed from the Bio Analytical Research Corporation South Africa PTY (LTD), Central Laboratory for Clinical Trials.

### Study measures

#### Demographic information

Data were collected on sex, age, race, nationality, ethnic group, marital status, highest level of education, and source of income.

#### Health profile and screening results

In addition to the health screening results of HIV status and self-reported antiretroviral therapy (ART) use, we report the following results:

*High-risk HPV*. Results include *any HR-HPV* (having at least one HR-HPV type), *Type 16*, *Type 18*, *other HR-HPV* (type 31, 33, 35, 39, 45, 51, 52, 56, 58, 59, 68, and 70; i.e.; having at least one of these HR-HPV types that is not HR-16 or HR-18), or *negative* (for any HR-HPV type). A participant could be co-infected with different types. HPV types were run for participants who consented for this screening, regardless of cervical cancer screening results.

*Cervical cancer screening*. Pap smear cytology results for cervical cancer screening could be one of the following–normal, atypical squamous epithelial cells of undetermined significance (ASCUS), low-grade squamous intraepithelial lesion (LSIL), or high-grade squamous intraepithelial lesion (HSIL). ASCUS, LSIL and HSIL were abnormal results. Women who were screened to have ASCUS (and/or HR-HPV) were recommended to have follow-up cervical cancer screening in twelve-months. Women found to have LSIL and HSIL were referred out to the Bara Gynaecology Department for further evaluation and management, if required.

*Previous cervical cancer screening*. Women were asked how many Pap smears they have undergone in their lifetime. Responses were categorised into *none*, *one*, *two*, *three*, or *more than three*.

*Newly diagnosed*. Women who did not self-report as knowing they were HIV-positive prior to HIV testing at the clinic.

*Previously diagnosed*. Women who self-reported as previously knowing they were HIV-positive prior to HIV testing at the clinic.

There was no HIV viral load data available for inclusion within this study.

### Data analysis

Frequencies and percentages were determined for categorical variables and stratified by age group (18–29, 30+ years), HIV status, ART use, and Pap smear results (normal and abnormal). To test for statistical significance for categorical measures stratified by age group and Pap smear results, and Chi-square analysis or Fisher’s exact test was used as appropriate. A global p-value was first determined and where this was significant, chi-square test of proportions at each level of a factor by HIV status, age-group or Pap smear results were conducted. Descriptive statistics such as medians and interquartile ranges were determined for continuous measures.

Since there is evidence in our data that HIV prevalence was higher in older women, comparisons of health screenings and HIV status controlling for age was assessed using logistic regression.

All statistical analyses were conducted in SAS Enterprise Guide 7.1 (SAS Institute, Cary, NC) using SAS/STAT procedures PROC FREQ and PROC MEANS.

## Results

### Demographics of participants by age group

Of the 513 women entering the clinic, 233 (45.4%) of the women opted out of the endocervical screening procedures. Therefore, the analysis included the remaining 280 women who did participate in the screening and allowed the use of their data. Of the 280 women, the majority proportion were black Africans (98.6%, [n = 276/280]), from South Africa (98.2%, [n = 275/280]), who were Zulu speaking (48.6%, [n = 135/278]), single (75.7%, [n = 212/280]), matriculated from high school (40.0%, [n = 112/280]), and employed or self-employed (51.8%, [n = 144/278]). The median age was 31 (interquartile range [IQR]: 25–40) years, with 57.9% (n = 162/280) of the women aged 30 or more years. (Table 1).

**Table 1:** Characteristics by age group of participants attending integrated HTS clinic in Soweto, South Africa; 18 June 2018–28 March 2019.

Variable	Total (N = 280)	18–29 years (n = 118)	≥30 years (n = 162)	P-Value
**Race**				
Black African	276/280 (98.6	116/118 (98.3)	160/162 (98.8)	0.9999
Coloured/mixed race	4/280 (1.4)	2/118 (1.7)	2/162 (1.2)	
**Nationality**				
Other	5/280 (1.8)	0/118 (0.00)	5/162 (3.1)	-
South African	275/280 (98.2)	118/118 (100.0)	157/162 (96.9)	
**Ethnic group**				
Other	21/278 (7.6)	7/118 (5.9)	14/160 (8.8)	0.4959
Sotho	51/278 (18.4)	25/118 (21.2)	26/160 (16.3)	
Tsonga	22/278 (7.9)	6/118 (5.1)	16/160 (10.0)	
Tswana	21/278 (7.6)	8/118 (6.8)	13/160 (8.1)	
Xhosa	28/278 (10.1)	11/118 (9.3)	17/160 (10.6)	
Zulu	135/278 (48.6)	61/118 (51.7)	74/160 (46.3)	
**Marital status**				
Divorced\widowed	9/280 (3.2)	0/118 (0.0)	9/162 (5.6)	-
Living Together\married	59/280 (21.1)	13/118 (11.0)	46/162 (28.4)	**0.0004**
Single	212/280 (75.7)	105/118 (89.0)	107/162 (66.1)	**<0.0001**
**Highest level of education attended**				
Matriculated	112/280 (40.0)	54/118 (45.8)	58/162 (35.8)	0.1071
Tertiary	58/280 (20.7)	26/118 (22.0)	32/162 (19.8)	
Up To High School	110/280 (39.3)	38/118 (32.2)	72/162 (44.4)	
**Source of money to live on**				
Employed/Self-Employed	144/278 (51.8)	37/118 (31.4)	107/160 (66.9)	**<0.0001**
Parents	54/278 (19.4)	44/118 (37.3)	10/160 (6.3)	**<0.0001**
Social Grant/Pensions	52/278 (18.7)	26/118 (22.0)	26/160 (16.3)	0.2216
Unemployed	28/278 (10.1)	11/118 (9.3)	17/160 (10.6)	0.7213

### Health screening results

**Fig 1:**
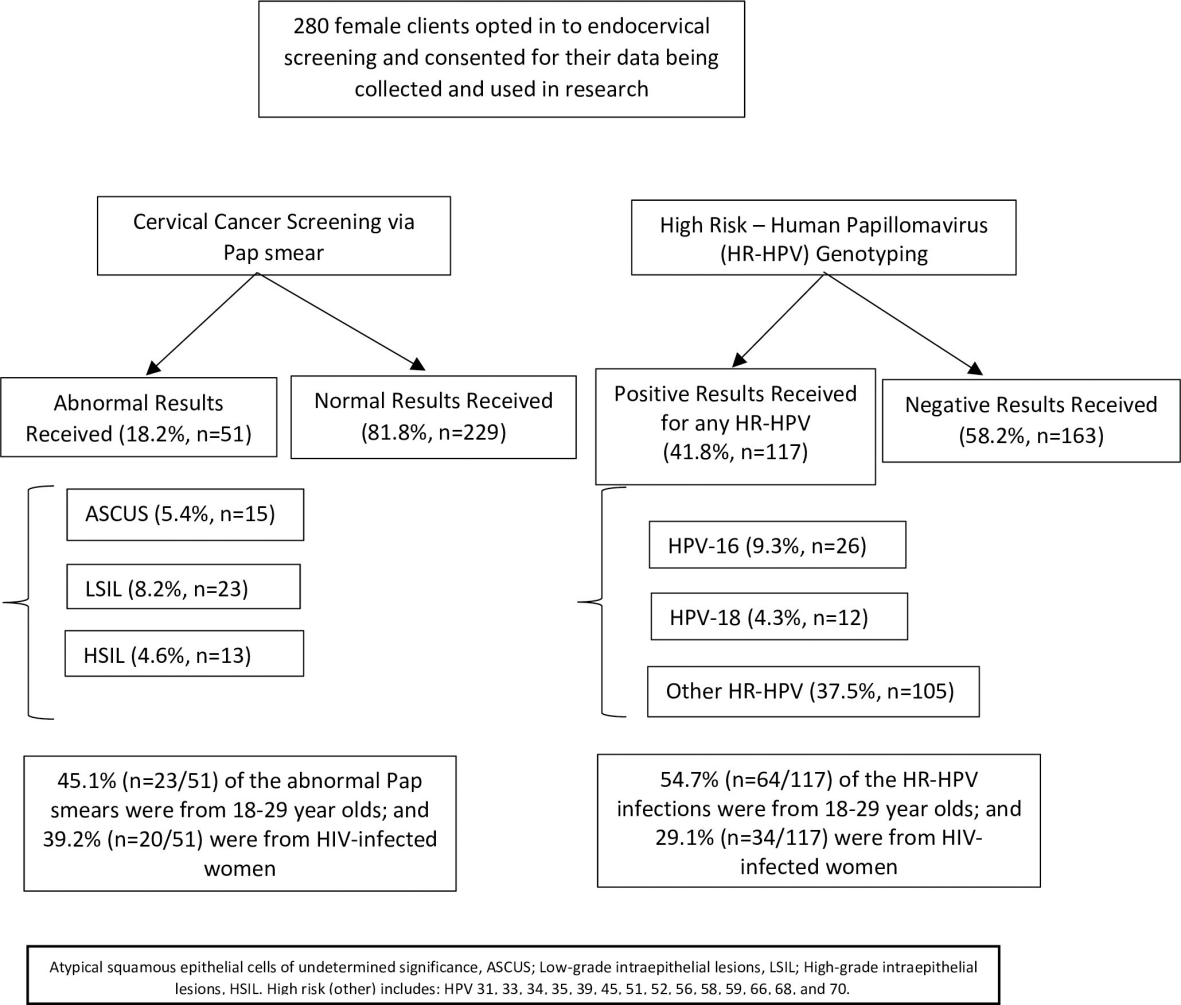
Female HTS client flow for participation in cervical cancer and HPV screening in Soweto, South Africa; 18 June 2018–28 March 2019.

Fig 1 depicts the proportions of abnormal cervical cancer screening results and positive HPV genotyping results by age group and HIV-status. Of all the women, 18.2% (n = 51/280) received an abnormal Pap smear result and 41.8% (n = 117/280) had at least one type of HR-HPV.

#### Health screening results by age group and HIV status

Table 2 depicts the health screening results stratified by age group and HIV status when controlling for ART use and age.

**Table 2:** Screening results by age-group and HIV status of women attending integrated HTS in Soweto, South Africa; 18 June 2018–28 March 2019.

Variable	Total (N = 280)	18–29 years (n = 118)	≥30 years (n = 162)	P-Value	HIV-positive^i^ (n = 57)	HIV-negative (n = 223)	P-value
**HIV Results**							
Positive	57/280 (20.4)	13/118 (11.0)	44/162 (27.2)	**0.0009**	--	--	--
Negative	223/280 (79.6)	105/118 (89.0)	118/162 (72.8)		--	--	
**Pap Smear**^**a**^							
Normal	229/280 (81.8)	95/118 (80.5)	134/162 (82.7)	0.6365	37/57 (64.9)	192/223 (86.1)	**0.0002**
ASCUS	15/280 (5.4)	4/118 (3.4)	11/162 (6.8)	0.2121	6/57 (10.5)^j^	9/223 (4.0)	0.0521
LSIL	23/280 (8.2)	15/118 (12.7)^b^	8/162 (4.9)	**0.0193**	8/57 (14.0)^j^	15/223 (6.7)	0.0729
HSIL	13/280 (4.6)	4/118 (3.4)^c^	9/162 (5.6)	0.3951	6/57 (10.5)^j^	7/223 (3.1)	**0.0180**
**How many Pap smears have you had in your lifetime?**^**d**^							
None	183/280 (65.4)	105/118 (89.0)	78/162 (48.2)	**<0.0001**	24/57 (42.1)^k^	159/223 (71.3)	**<0.0001**
One	69/280 (24.6)	10/118 (8.5)	59/162 (36.4)	**<0.0001**	23/57 (40.4)	46/223 (20.6)	**0.0020**
Two	12/280 (4.3)	2/118 (1.7)	10/162 (6.2)	0.0677	3/57 (5.3)	9/223 (4.0)	0.6831
Three	10/280 (3.6)	1/118 (0.9)	9/162 (5.6)	**0.0361**	5/57 (8.8)	5/223 (2.2)	**0.0178**
More than three	6/280 (2.1)	0/118 (0.0)	6/162 (3.7)	-	2/57 (3.5)	4/223 (1.8)	0.4249
**Frequency of HR-HPV infections**							
Any HR-HPV (other)^e^	105/280 (37.5)	58/118 (49.2)	47/162 (29.0)	**0.0006**	32/57 (56.1)	73/223 (32.7)	**0.0011**
HPV-16	26/280 (9.3)	14/118 (11.9)	12/162 (7.4)	0.2045	8/57 (14.0)	18/223 (8.1)	0.1662
HPV-18	12/280 (4.3)	7/118 (5.9)	5/162 (3.1)	0.2457	2/57 (3.5)	10/223 (4.5)	0.7455
HPV-16 or HPV-18^f^	38/280 (13.57)	21/118 (17.80)	17/162 (10.49)	0.0781	10/57 (17.54)	28/223 (12.56)	0.3265
Any HR-HPV^g^	117/280 (41.8)	64/118 (54.2)	53/162 (32.7)	**0.0003**	34/57 (59.7)	83/223 (37.2)	**0.0022**
No HR-HPV	163/280 (58.2)	54/118 (45.8)	109/162 (67.3)	**0.0003**	23/57 (40.4)	140/223 (62.8)	**0.0022**
**HPV Serotypes**^**h**^							
Only infected with HR (other)	79/280 (28.2)	43/118 (36.4)	36/162 (22.2)	**0.0090**	24/57 (42.1)	55/223 (24.7)	**0.0090**
Only infected with HPV-16	8/280 (2.9)	3/118 (2.5)	5/162 (3.1)	0.9999	1/57 (1.8)	7/223 (3.1)	0.9999
Only infected with HPV-18	4/280 (1.4)	3/118 (2.5)	1/162 (0.6)	0.3134	1/57 (1.8)	3/223 (1.4)	0.9999
HR (other) + HPV-16 coinfection	18/280 (6.4)	11/118 (9.3)	7/162 (4.3)	0.0920	7/57 (12.3)	11/223 (4.9)	**0.0435**
HR (other) + HPV-18 coinfection	8/280 (2.9)	4/118 (3.4)	4/162 (2.5)	0.7247	1/57 (1.8)	7/223 (3.1)	0.9999

Atypical squamous epithelial cells of undetermined significance, ASCUS; Human Papilloma Virus, HPV; High-risk, HR; High grade squamous intraepithelial lesion, HSIL; Low grade squamous intraepithelial lesion, LSIL; Low grade squamous intraepithelial lesion.

^a^ HIV-uninfected women aged <30 years had significantly more abnormal pap smear results than HIV-uninfected women aged 30+ years (9%, n = 20/105 vs 9% n = 11/118; p = 0.0361).

^b^ Of the 15 younger women with LSIL, 12 (80.0%) were HIV-negative.

^c^ All 4 younger women with HSIL were HIV-negative.

^d^ Of the 11% (n = 13/118) of younger women who have had a pap smear in their lifetime, 7 (53.8%) were HIV-positive.

^e^ High risk (other) includes: HPV 31, 33, 34, 35, 39, 45, 51, 52, 56, 58, 59, 66, 68, and 70.

^f^There were no women infected with both HPV-16 and HPV-18.

^g^ Of the 117 HR-HPV-infected women, 22.2% (n = 26/117) had either HPV-16 or HPV-18 as well as at least one other HR-HPV type, 54.7% (n = 64/117) were amongst 18–29 year olds, and 29.1% (n = 34/117) were HIV-positive.

^h^ HPV Serotypes are mutually exclusive categories.

^i^ Of the 57 HIV-infected participants, 54.4% (n = 31/57) were newly diagnosed with HIV and 45.6% (n = 26/57) already knew their status.

^j^ Of the 20 HIV-positive women with abnormal Pap smear, 55.0% (11/20) were newly diagnosed with HIV.

^k^ Of the HIV infected women, older women (30+ years) were less likely to have never had a pap smear compared to young women (40.9% [n = 18/44] vs. 46.2% [n = 6/13]; p = 0.7365).

Overall, 20.4% (n = 57/280) of the women were HIV-positive, with women at least 30 years of age having significantly more HIV-infection than the younger age group (27.2% [n = 44/162] vs 11.0% [n = 13/118]; p = 0.0009).

Of the Pap smear results, 18.2% (n = 51/280) were abnormal; and of those, 29.4% (n = 15/51) had ASCUS, 45.1% (n = 23/51) had LSIL and 25.5% (n = 13/51) had HSIL. Of the abnormal Pap smears, 45.1% (n = 23/51) were from 18–29 year olds, of which, 87.0% (n = 20/23) were HIV-uninfected; and 39.2% (n = 20/51) were from HIV-infected women. All four 18–29 year olds found to have HSIL were HIV-uninfected.

HIV-positive women were more likely to have an abnormal cytology result as compared to HIV-negative women (35.1%, [n = 20/57] vs. 13.9%, [n = 31/223]; p = 0.0002) and specifically a higher proportion of HSIL (10.5%, [n = 6/57] vs. 3.1%, [n = 7/223]; p = 0.028). The younger women had significantly more LSIL than the older women (12.7% [n = 15/118] vs 4.9% [n = 8/162]; p = 0.019).

Overall, the majority of women self-reported having never had a Pap smear in their lifetime (65.4% [n = 183/280]), with only 51.9% (n = 84/162) and 57.9% (n = 33/57) of women over 30 years of age or with HIV-infection having ever had a Pap smear, respectively. While not significant, HIV-positive older women (30+ years) were less likely to have never had a Pap smear compared to younger HIV-positive women (40.9% [n = 18/44] vs. 46.2% [n = 6/13]; p = 0.7365). Of the nine women aged 30+ years with HSIL, five (55.6%) had never had a pap smear previously and six (66.7%) were HIV-infected. Of the thirteen HIV-positive 18–29 year olds, seven 53.8% had a pap smear previously.

Of the women who were screened for HR-HPV, 41.8% (n = 117/280) were infected with at least one type, of which 54.7% (n = 64/117) were from 18–29 year olds and 29.1% (n = 34/117) were from HIV-positive women. Of these, the majority were infected with other HR-HPV type(s) (89.7%, [n = 105/117]). However, 22.2% of HR-HPV-infected women (n = 26/117) had either HPV-16 or HPV-18 as well as at least one other HR-HPV type. None of the women had co-infection with HPV-16 and HPV-18.

Women who were 18–29 years of age had significantly more other HR-HPV types (49.2% [n = 58/118] vs 29.0% [n = 47/162]; p = 0.0006) and any HR-HPV (54.2% [n = 64/118] vs 32.7% [n = 53/162]; p = 0.0003) than woman who were at least 30 years of age. HIV-positive women had significantly more HR-HPV infection (59.6% [n = 34/57] vs 37.2% [n = 83/223]; p = 0.0003) than the HIV-negative women. Specifically, HIV-positive women had significantly more other HR-HPV types (56.1% [n = 32/57] vs 32.7% [n = 73/223]; p = 0.0002) and co-infection with other HR-HPV type and HPV-16 (12.3% [n = 7/57] vs 4.9% [n = 11/223]; p = 0.025) than their HIV-negative counterparts.

#### Health screening results by HIV status controlling for age and ART use

Of the 57 HIV-positive women, 45.6% (n = 26) already knew their HIV status, of which 69.2% (n = 18) self-reported they were not on ART and 34.6% (n = 9) reported never having a Pap smear previously. Over half (55.0%, n = 11/20) of the HIV-positive women who screened with an abnormal Pap smear were newly diagnosed with HIV.

Though not significant, a high proportion of those with abnormal Pap smear and other HR-HPV high risk types were on ART compared to those not on ART (38.9%[n = 7/18] vs. 25.0%[n = 2/8]; p = 0.667 and 55.6%[n = 10/18] vs. 12.5%[n = 1/8]; p = 0.084; respectively). Additionally, a high proportion of HIV-positive women with HPV-16 were not on ART compared to HIV-positive women with HPV-16 on ART (12.5%[n = 1/8] vs. 5.6%[n = 1/18]; p = 0.529). None of the HIV-positive women had HPV-18.

#### Abnormal cervical cancer screening and HR-HPV co-infection

Table 3 shows that women with abnormal Pap smear results were more likely to have any HR-HPV infections (84.3% [n = 43/51] vs 32.3% [n = 74/229]; p<0.0001), other HR-HPV types (80.4% [n = 41/51] vs 27.9% [n = 64/229]; p<0.0001), HPV-16 (27.5% [n = 14/51] vs 5.2% [n = 12/229]; p<0.0001), and co-infection of the two (23.5% [n = 12/51] vs 2.6% [n = 6/229]; p<0.0001) than women with normal Pap smear results.

**Table 3:** HPV serotypes by pap smear results of women attending integrated HTS in Soweto, South Africa; 18 June 2018–28 March 2019.

Variable	Total	Abnormal (n = 51)^a^	Normal (n = 229)	P-Value
**Frequency of HR-HPV infections**				
Any HR (other)^b^	105/280 (37.5)	41/51 (80.4)	64/229 (28.0)	**<0.0001**
HPV-16	26/280 (9.3)	14/51 (27.5)	12/229 (5.2)	**<0.0001**
HPV-18	12/280 (4.3)	2/51 (3.9)	10/229 (4.4)	0.8871
HPV-16 or HPV-18^c^	38/280 (13.57)	16/51 (31.37)	22/229 (9.61)	**<0.0001**
Any HR-HPV	117/280 (41.8)	43/51 (84.3)	74/229 (32.3)^e^	**<0.0001**
**HPV Serotypes**^**d**^				
Only infected with HR (other)	79/280 (28.2)	27/51 (52.9)	52/229 (22.7)	**<0.0001**
Only infected with HPV-16	8/280 (2.9)	2/51 (3.9)	6/229 (2.6)	0.6404
Only infected with HPV-18	4/280 (1.4)	0/51 (0.0)	4/229 (1.8)	-
HR (other) + HPV-16 coinfection	18/280 (6.4)	12/51 (23.5)	6/229 (2.6)	**<0.0001**
HR (other) + HPV-18 coinfection	8/280 (2.9)	2/51 (3.9)	6/229 (2.6)	0.6404
No HR-HPV	163/280 (58.2)	8/51 (15.7)	155/229 (67.7)	**<0.0001**

Human Papilloma Virus, HPV.

^a^Abnormal Pap smear results include atypical squamous epithelial cells of undetermined significance (ASCUS), low-grade squamous intraepithelial lesions (LSIL), and high-grade squamous intraepithelial lesions (HSIL).

^b^High risk (other) includes: HPV 31, 33, 34, 35, 39, 45, 51, 52, 56, 58, 59, 66, 68, and 70.

^c^There were no women infected with both HPV-16 and HPV-18.

^d^HPV Serotypes are mutually exclusive categories.

^e^Of the 74 women with any HR-HPV and normal Pap smear, 23% (n = 17/74) and 77% (n = 57/74) were HIV-infected and HIV uninfected, respectively.

## Discussion

Of a primarily black African adult female population from a peri-urban setting in South Africa, one-fifth of the women were HIV-positive, nearly one-in-five women had an abnormal Pap smear, and over two-fifths had at least one HR-HPV infection. Overall, cervical cancer screening coverage was low, with only two-thirds of all women having been screened previously and only half of both women over 30 years of age or with HIV-infection having ever had a Pap smear in their lifetime. Of the abnormal Pap smears, about half were from 18–29 year olds; of which all with HSIL and four-fifths with LSIL were HIV-negative and would have not been eligible for cervical cancer screening according to the national guidelines, if asymptomatic. Over half of HR-HPV infections were amongst younger women, with nearly one-in-five 18–29 year olds infected with either HPV-16 or HPV-18. Nearly one-third of HR-HPV infections were from HIV-positive women, and one-third of women with normal Pap smears remained with HR-HPV infection. These study findings may provide evidence to support a change in the type of cervical cancer prevention and/or screening mechanism used and age at which screening begins.

Despite the long-term existence of the national cervical cancer screening programme screening low-risk women from 30 years of age and high-risk HIV-positive women from diagnosis, our study findings show the programme is still producing low screening coverage rates. Of our study participants who were eligible for cervical cancer screening based on age or HIV-status, just over half (55.4%) had ever been screened. While this coverage is more promising than the National District Health Information System data collated in 2019, which suggested less than one-fifth of eligible women received screening [1], it is still concerning, especially when also considering the non-eligible women identified within our study as being at-risk.

Younger women under 30 years of age, if asymptomatic and HIV-negative (which 89% were), represent a significant challenge–at high-risk for HPV infection, too old to have been included in the HPV vaccination campaign targeting 9–12 year old girls, and yet not eligible for Pap smear screening within the existing screening programme due to their younger age [11]. Of all the abnormal Pap smear results, half were amongst our young participants–nearly all of which were pre-cancerous lesions and amongst HIV-negative women. Likewise, over half of the women with HR-HPV infections were 18–29 year olds, of which one-in-five were infected with at least HPV-16 or HPV-18—the types holding the highest risk for cervical intraepithelial neoplasia 3 and occult cancer [18]. Our findings corroborated with a study conducted within five Gauteng primary healthcare facilities on the peak prevalence of LSIL amongst the younger age group (<25 years), likely demonstrating the previously established correlation between LSIL and recent HPV infection [19, 20].

Furthermore, HIV-positive women who do not yet know their status and are asymptomatic are also included within the SANDoH cervical cancer screening guidelines’ low-risk structured screening population [11]. Therefore, standard screening at ten-year intervals, only beginning at 30 years of age, would apply. Compounding this, is that national peak HIV infections occur in young women aged 15–24 years [21], comprising 37% of new infections documented in 2016 [22]. Demographic and Health Surveys from 19 LMICs mostly within sub-Saharan Africa project about half of youths aged 15–19 years, about one-quarter (24%) of young adults aged 20–24 years, and 22% of adults aged 25–29 years do not know they are living with HIV [23]. Our data show over two-fifths of our HIV-positive women had never had a Pap smear, of which one-quarter were under 30 years of age and three-quarters were 30+ years of age. Only half of these women already knew their HIV status.

Lastly, about one-third of women with normal cytology still had HR-HPV infections. The American Society for Colposcopy and Cervical Pathology’s (ASCCP) risk-based management guidelines suggest that additional evaluation (e.g., colposcopy with biopsy) is necessary even when cytology results are negative [18], and surveillance with cytology, alone, is only acceptable if HPV screening or co-testing is not feasible [18]. The current South African Cervical Cancer Screening Guidelines suggest HPV testing where resources allow, and the HPV Advisory Board recommends the commencement of such screening at an earlier age of 25 years among HIV-negative women [14]. The results of this study can be used to inform discussions by stakeholders about efforts to reduce HPV, including the HPV Advisory Board’s recommendation to lower the screening age to 25 years [15].

The Roche Linear Array, as used in this study for HPV genotyping, is not yet an approved primary screening test in South Africa, despite studies reporting it fulfills the clinical accuracy requirements to detect cervical intraepithelial neoplasia of grade 2 or worse (CIN2 +) [24–26]. Given that evidence has shown that HPV-based screening provides 60–70% greater protection against invasive cervical carcinomas compared with cytology [27] and is more cost-effective in the long-term [28], additional research is needed to determine other practical implementation impacts of changing from cytology- to HPV-based primary screening for cervical cancer (i.e.; financial, operational, etc.) amongst LMICs. However, given the high prevalence of HPV, the implementation of follow-ups for all HR-HPV-infected women could potentially add pressure on an already burdened primary healthcare system [29]. With other HR-HPV types making up the vast majority of infections in our study, there may be a more urgent need for clinical research on a larger valent (e.g.; 9-valent) vaccine product. While the Cervarix vaccine used by the nationwide vaccination programme protects more widely than just HPV-16 and 18 in other country populations [30], it does not necessarily reduce the need for a nonavalent vaccine.

### Limitations

This study included a convenience sample of participants self-selecting to attend a single HTS clinic in Soweto. There is potential for bias as these participants have not been randomly sampled from multiple facilities. Overall, our cohort had a small sample size. Some of our sub-group sample sizes for comparison are even smaller, which may have affected statistical precision.

The integrated HTS clinic operated within a research unit, and is not a government health centre. This impacts generalisability to typical community health clinic settings. Previous conduct of Pap smears and HIV testing, as well as ART use, was also self-reported by the HTS clients and not verified by study staff. The HPV genotyping results could not be disaggregated further into nonavalent and valent types, which may have limited interpretation of clinical significance.

### Conclusion

In recognizing the current burden of cervical cancer amongst the general population of women, its anticipated impact on HIV-positive women, and the current gaps within the national cervical cancer screening policy, it is critically important to pursue models of integrated services that effectively prevent, diagnose, treat, and control cervical cancer. In addition to an expansion in age range of the HPV vaccination programme [31] and lowering the age for cervical cancer screening for women deemed low-risk, our findings suggest opportunities for additional research to study potential outcomes accompanying changes in the types of prevention and/or screening mechanism used and age at which screen begins. Stakeholders may want to consider ways to provide cervical cancer screening for women who test HIV-positive and/or with HR-HPV infection, which may lead to early detection [16].

## Supporting information

S1 Dataset (XLSX)
